# Making randomised trials more efficient: report of the first meeting to discuss the Trial Forge platform

**DOI:** 10.1186/s13063-015-0776-0

**Published:** 2015-06-05

**Authors:** Shaun Treweek, Doug G. Altman, Peter Bower, Marion Campbell, Iain Chalmers, Seonaidh Cotton, Peter Craig, David Crosby, Peter Davidson, Declan Devane, Lelia Duley, Janet Dunn, Diana Elbourne, Barbara Farrell, Carrol Gamble, Katie Gillies, Kerry Hood, Trudie Lang, Roberta Littleford, Kirsty Loudon, Alison McDonald, Gladys McPherson, Annmarie Nelson, John Norrie, Craig Ramsay, Peter Sandercock, Daniel R Shanahan, William Summerskill, Matt Sydes, Paula Williamson, Mike Clarke

**Affiliations:** Health Services Research Unit, University of Aberdeen, Aberdeen, AB25 2ZD UK; Centre for Statistics in Medicine, Nuffield Department of Orthopaedics, Rheumatology, and Musculoskeletal Sciences, University of Oxford, Botnar Research Centre, Nuffield Orthopaedics Centre, Windmill Road, Oxford, OX3 7LD UK; Medical Research Council North West Hub for Trials Methodology Research, Manchester Academic Health Science Centre, Centre for Primary Care, University of Manchester, Oxford Road, Manchester, M13 9PL UK; James Lind Initiative, Oxford, UK; MRC/CSO Social and Public Health Sciences Unit, University of Glasgow, 200 Renfield Street, Glasgow, G2 3QB UK; Medical Research Council, Methodology Research Programme (MRC MRP), London, UK; Consultant in Public Health and Head of Health Technology Assessment, National Institute for Health Research, Evaluation, Trials, and Studies Coordinating Centre, University of Southampton, Alpha House, Enterprise Road, Southampton, SO16 7NS UK; School of Nursing and Midwifery, National University of Ireland Galway, University Road, Galway, Ireland; Nottingham Clinical Trials Unit (NCTU), Nottingham Health Science Partners, C Floor, South Block, Queens Medical Centre, Derby Road, Nottingham, NG7 2UH UK; Warwick Medical School, The University of Warwick, Coventry, CV4 7AL UK; London School of Hygiene & Tropical Medicine, Keppel Street, London, WC1E 7HT UK; National Perinatal Epidemiology Unit, University of Oxford, Oxford, UK; North West Hub for Trials Methodology Research, University of Liverpool, 1st floor Duncan Building, Daulby Street, Liverpool, L69 3GA UK; South East Wales Trials Unit (SEWTU), School of Medicine, Cardiff University, Cardiff, UK; The Global Health Network, Oxford University Centre for Tropical Medicine, University of Oxford, Oxford, UK; Tayside Clinical Trials Unit, University of Dundee, Dundee, UK; Marie Curie Palliative Care Research Centre, Cardiff University School of Medicine, Heath Park, Cardiff, Wales, CF14 4YS UK; Division of Clinical Neurosciences, The University of Edinburgh, Western General Hospital, Crewe Road, Edinburgh, EH4 2XU UK; BioMed Central Ltd, 236 Gray’s Inn Road, London, WC1X 8HB UK; The Lancet, London, UK; Medical Research Council, Clinical Trials Unit (MRC CTU), London, UK; North West Hub for Trials Methodology Research and Department of Biostatistics, University of Liverpool, 1st floor Duncan Building Daulby Street, Liverpool, L69 3GA UK; Institute of Clinical Sciences, Block B, Queens University Belfast, Royal Victoria Hospital, Grosvenor Road, Belfast, BT12 6BA UK

**Keywords:** Randomised controlled trials, methodology, efficiency, research waste

## Abstract

Randomised trials are at the heart of evidence-based healthcare, but the methods and infrastructure for conducting these sometimes complex studies are largely evidence free. Trial Forge (www.trialforge.org) is an initiative that aims to increase the evidence base for trial decision making and, in doing so, to improve trial efficiency.

This paper summarises a one-day workshop held in Edinburgh on 10 July 2014 to discuss Trial Forge and how to advance this initiative. We first outline the problem of inefficiency in randomised trials and go on to describe Trial Forge. We present participants’ views on the processes in the life of a randomised trial that should be covered by Trial Forge.

General support existed at the workshop for the Trial Forge approach to increase the evidence base for making randomised trial decisions and for improving trial efficiency. Agreed upon key processes included choosing the right research question; logistical planning for delivery, training of staff, recruitment, and retention; data management and dissemination; and close down. The process of linking to existing initiatives where possible was considered crucial. Trial Forge will not be a guideline or a checklist but a ‘go to’ website for research on randomised trials methods, with a linked programme of applied methodology research, coupled to an effective evidence-dissemination process. Moreover, it will support an informal network of interested trialists who meet virtually (online) and occasionally in person to build capacity and knowledge in the design and conduct of efficient randomised trials.

Some of the resources invested in randomised trials are wasted because of limited evidence upon which to base many aspects of design, conduct, analysis, and reporting of clinical trials. Trial Forge will help to address this lack of evidence.

## Background

‘*There is a peculiar paradox that exists in trial execution* - *we perform clinical trials to generate evidence to improve patient outcomes*; *however*, *we conduct clinical trials like anecdotal medicine*: (*1*) *we do what we think works*; (*2*) *we rely on experience and judgement and* (*3*) *limited data to support best practices*.’

Monica Shah, quoted in Gheorghiade *et al*. [1].

This paper summarises a one-day workshop held in Edinburgh on 10 July 2014 to discuss Trial Forge (www.trialforge.org), an initiative focused on improving randomised trial efficiency and quality. The initiative is aimed at the people who design and run trials, staff at trials units, for example, or clinicians and others who design studies. In this paper, we outline the problem of inefficiency in trials and describe the Trial Forge initiative to improve efficiency. We hope that many of those reading the paper will be interested in contributing to Trial Forge in the future.

Randomised trials (hereafter ‘trials’), especially when brought together in systematic reviews, are regarded as the gold standard for evaluating the effects of healthcare treatments, with thousands of trials and hundreds of systematic reviews reported every year. PubMed has indexed over 370,000 reports of randomised trials; the World Health Organisation’s International Clinical Trials Registry Platform [2] contains over 250,000 trial records, of which, 71,000 are listed as recruiting; and the Cochrane Central Register of Controlled Trials contains more than 800,000 records. Tens of billions of dollars of public and private money are invested globally in trials every year (US $25 billion in the United States alone in 2010 [3]) and the average cost of a trial per participant is estimated to be almost £8,500 in the United Kingdom [4].

Many of these resource are wasted, often because insufficient account is taken of existing evidence when choosing questions to address [5], and results are either not published or poorly reported. Moreover, despite trials being a cornerstone of evidence-based healthcare, the methods and infrastructure for conducting these complex studies are largely evidence free [6]. For example, every trial has to recruit and retain participants, but only a handful of recruitment and retention strategies and interventions are currently supported by high-quality evidence [7, 8]. A recent analysis found that only 55 % of UK National Institute of Health Research and Medical Research Council (MRC) trials (a set of large, relatively well-funded studies in the UK) recruiting between 2002 and 2008 met their recruitment targets [9]. The same study found that extensions are common, with 45 % of trials needing at least one funding extension, although only 55 % of these then go on to meet their recruitment targets. Furthermore, although data collection is central to trials and can consume a large proportion of trial resources, researchers often collect more data than they are able to analyse and publish [10]. Indeed, there is a dearth of research into the optimal methods for data collection and data management [11]. This is a different problem from selective reporting, where bias is introduced through the way outcomes are selected and presented in trial reports, especially for harms [12]. Vera-Badillo and colleagues called this type of bias ‘spin’ [13].

As a consequence, the most appropriate methods are not always chosen when trials are designed, leading to trial management and delivery problems later. Indeed, poor design decisions may do more than make a trial difficult to deliver; they may mean that any eventual findings will be of limited value. This could be because, for example, the comparator used renders the trial clinically irrelevant [14], the outcome measures are not relevant to those making treatment decisions [15], or the patients involved do not represent the majority of patients with the condition of interest [16]. The patients, health professionals, and policymakers who look to systematic reviews of trials for help in their decision making are often frustrated to find that the questions addressed by researchers do not reflect clinical decision making needs (a failure of prioritisation) [17], have dubious relevance in their settings [17–19], or that failings in the conduct or reporting of trials mean that they do not provide the reliable and robust evidence that they need. Some trials may simply be unnecessary [20]. This all represents an unacceptably wasteful approach to designing, running, analysing, and reporting trials. The problem of inefficiency in medical research is not new: Schwartz and Lellouch urged trialists to change the way they designed trials as long ago as 1967 [21], Altman pointed to the scandal of poor medical research in 1994 [22], and, in 2009 [23], Chalmers and Glasziou estimated that more than 85 % of the resources invested in medical research was being avoidably wasted. What has been lacking is a coordinated attempt to tackle inefficiency in clinical trials.

## Main text

### Trial Forge

Trial Forge (www.trialforge.org) aims to address the lack of an accessible evidence base around trial efficiency and quality. A one-day workshop, funded by the Network of MRC Hubs for Trials Methodology Research and the Health Services Research Unit at the University of Aberdeen, UK, was held in Edinburgh on 10 July 2014 to discuss the initiative. The grant holders of the MRC Hub funding (Marion Campbell, Mike Clarke, Athene Lane, Trudie Lang, John Norrie, Shaun Treweek, and Paula Williamson) invited 38 participants with experience in methodology and trial design, trial management, statistics, data management, clinical care, commissioning and publishing research, public and patient involvement, and providing trial support through trials units to the worship.

The aims of the workshop were as follows:

1. To share knowledge on resources that already exist with regard to efficient trials.
2. To share knowledge on guidance relating to trial design, conduct, analysis, and reporting.
3. To agree on the key processes of the trial pathway, that is, the major processes in the life of a trial.
4. To begin to suggest features that Trial Forge must have.
5. To promote awareness of Trial Forge.
6. To produce a statement paper on the Trial Forge initiative

As the workshop members were professional trialists, trial managers, statisticians, and others involved in trial design, conduct, analysis, and reporting and the discussions were of current practice, no formal ethics approval, or consent was deemed necessary.

### How will Trial Forge work?

Discussion at the workshop highlighted several substantial problems, some of which are listed in Table 1. Trial Forge aims to remove or reduce these problems and others through targeted collaborative work. Some of the ways it will do this are listed in Table 1. Trial Forge will use a five-step process to identify and address gaps in knowledge about trial method:

**Table 1:** Trial Forge Examples of trial challenges and how Trial Forge could help

General problem	Examples	Examples of how Trial Forge aims to help
Information is spread over many journals, websites, books and other publications, which makes it difficult to access and use in decision making. This makes finding and navigating the literature time-consuming and challenging.	Searching Pubmed [http://www.ncbi.nlm.nih.gov/sites/entrez/, searched 2 Jan 2015] using the phrase *clinical trial recruitmen*t and limiting to reviews in the last 5 years produces 252 hits, too large a number to sift through easily.	To collate, or link people to, existing high-quality evidence on key trial processes. For recruitment this would include: what influences recruitment strategies that can improve recruitment how to tailor recruitment strategies to particular contexts
	A search on Google Scholar [http://scholar.google.com, searched 2 Jan 2015] using the same phrase (exact phrase search) produces 1080 hits since 2010.	To develop targeted research agendas designed to fill gaps in knowledge around how best to recruit trial participants.
	Searching Amazon [http://www.amazon.co.uk searched 2 Jan 2015] for *clinical trial recruitment* produces 525 hits; the first page results of includes books costing less than £1 to over £900.	To make it easier for teams to work together to address these research agendas.
		In the absence of high-quality evidence, provide a repository for the experience and knowledge from the community of trialists as to how they recruit participants.
There are substantial gaps in the evidence base for key issues that affect all trials and which are not being systematically targeted by methodology research.	There is little published research evidence to inform decisions about trial management options such as how best to select clinical sites, how to maintain relationships with sites, how to model the movement of patients and staff through trial processes, or how to effectively train trial and site staff.	To develop targeted research agendas designed to fill gaps in knowledge about how to design, run, analyse and report trials.
		For trial management, the development of methods to allow trial managers to share their solutions without the need for full publications, which are not generally part of the career development of trial managers (ie. there is no incentive to publish).
		Encourage systematic reviewers (eg. of Cochrane reviews) to suggest concrete methodological studies that need to be done and to link these to initiatives such as SWATs [43, 44] to provide ready-made protocols for those studies.
		Systematically direct information about evidence gaps to funding agencies for their consideration as part of their prioritisation process for the selection of topics for funding calls.
Much trial knowledge is tacit and held by experienced staff working at trials units, other similar centres, or on individual trials.	Although many research groups and units cost, manage and create data management systems for trials, there is little easily available information on effective ways of how to complete each of these processes.	In the absence of high-quality evidence, provide a repository for the experience and knowledge from the community of trialists as to how they design, run, analyse and report their trials.
		Collate and evaluate tools that are being used by groups designing and running trials such as trials units and other similar centres.
		To develop targeted research agendas designed to move from tacit, often unevaluated knowledge, to high-quality evaluated evidence.
There is no easy way for individuals needing advice to access it from the potentially thousands of people who have knowledge that might help them.	If a trial data management team using the OpenClinica system encounters a technical problem, there is an active online community that provides help free (https://community.openclinica.com). Questions are answered quickly. There are few similar opportunities to quickly address questions on trial design, conduct, analysis or reporting.	Provide a repository for the experience and knowledge from the community of trialists as to how they design, run, analyse and report their trials.
		Provide support for electronically linked communities of practice (e.g. through Question & Answer and discussion sections on its website)
		Learn from The Global Health Network (https://tghn.org) on how to build online communities in healthcare.
Information is not structured in a way that helps people find what they need to resolve their uncertainties. People working on trials have questions (such as ‘Should I visit the sites to boost recruitment?’, ‘How much quality assurance do I need to do?’, ‘Will adding an extra outcome measure affect recruitment and retention?’), but guidance is rarely organised around questions and the answers to them.	The Clinical Trials Toolkit (http://www.ct-toolkit.ac.uk/routemap) provides regulatory and other information about drug trials in the UK Although useful, the information is structured like a text book. People visiting the site, however, are likely to have done so because they have a series of questions about their trial and are looking for answers. The textbook structure makes answering these questions slower than it could be.	Provide a mixed structure to Trial Forge, where much of the material is directly framed as questions and answers. Where evidence provides a clear answer, this information will be presented as a question.
ᅟ	ᅟ	Work with trialists to present information in such a way that it enables them to find answers to their questions as quickly as possible.
There is no easy way to support collaborative, trial methodology research to address evidence gaps and shortcomings.	The 2010 Cochrane review of interventions to improve trial recruitment [7] includes 45 trials evaluating 46 interventions. Despite this, the review concludes that there is high-quality evidence supporting only three or so interventions. The effectiveness or otherwise of the other interventions remains unclear.	The initiatives listed above will help to identify gaps in evidence. Trial Forge will then highlight these, including to funders in an effort to focus researcher effort on important and known gaps.
		By supporting SWATs [43, 44], researchers wishing to fill at least some of these gaps will be able to use existing (and common) protocols to evaluate given interventions.
		Provide electronically linked communities that can agree to work together to fill a gap by, for example, evaluating the same intervention across many trials. A good example of this approach is the MRC START project for recruitment interventions: http://www.population-health.manchester.ac.uk/mrcstart/

1. Identify trial processes
2. Collate what is known about these processes.
3. Strengthen the evidence base by creating a methodology research agenda.
4. Collaborate to work through the methodology research agenda.
5. Dissemination.

#### Step 1 - Identify trial processes

Step 1 will identify the processes that make up a trial, starting with the main processes (for example, recruitment) and then breaking these down into smaller processes (for example, how to set the eligibility criteria for a trial, selecting the components of the recruitment strategy, identifying potential participants, and targeting appropriate recruitment strategies for them). This is similar to the process improvement approach taken by the British cycling team in its preparation for the 2012 London Olympic Games. Dave Brailsford, British Cycling's Performance Director at the time said when asked about the team’s approach:

‘*The whole principle came from the idea that if you broke down everything you could think of that goes into riding a bike*, *and then improved it by 1* %, *you will get a significant increase when you put them all together*.’ [24]

There are very many processes involved in a trial, and learning about, and improving each of them may have a minimal effect on its own, but taken together, these improvements could have a much more profound impact.

**Fig. 1:**
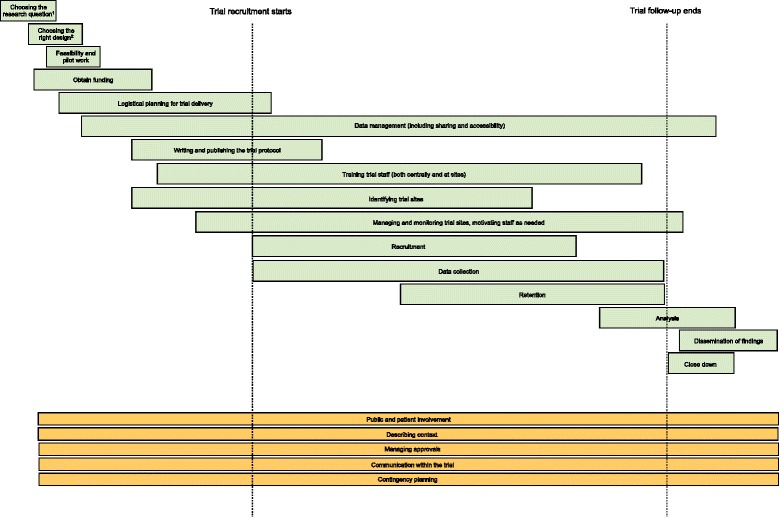
Key processes of the trial pathway (many of which are overlapping and non-linear). Suggestions from a one-day workshop held in Edinburgh on 10 July 2014. The placement and length of the bars gives an indication of when in the trial they start and end, though this is likely to vary greatly between trials

Participants at the Edinburgh workshop produced an initial list of headline trial processes (Fig. 1) for which collating (and creating) research evidence would be beneficial. This list will form the starting point for Trial Forge work.

#### Step 2 - Collate what is known about these processes

In Step 2, Trial Forge will either identify existing initiatives to collate what is known about individual processes or work to collate the evidence (which may include providing links to ongoing studies) and integrate reviews (and other relevant literature) using both quantitative and qualitative synthesis approaches [25–28]. For example, for help in choosing trial outcomes, Trial Forge would direct people towards the COMET (Core Outcome Measures in Effectiveness Trials [29], http://www.comet-initiative.org) Initiative. COMET has systematically reviewed published standardised core outcome sets for trials [30], and combined these in the COMET database with information on core outcome sets currently being developed. As another example, for help with choosing evidence-based recruitment interventions, the MRC Network of Hubs for Trials Methodology Research is funding a project to develop a searchable database containing published and ongoing research into recruitment. On a smaller scale, Cochrane Methodology Reviews, and other systematic reviews have brought together existing research in specific topic areas. These will be highlighted in Step 2. Epistemonikos (http://www.epistemonikos.org/en/), a website that links together systematic reviews, overviews of reviews, and primary studies to support health-policy decisions, is another example of how research evidence can be collated.

More generally, the Evidence-Based Research Network (http://www.ebrnetwork.org) is an example of an initiative that aims to promote the efficient use of existing research, especially through the use of systematic reviews [31] and information about ongoing research. Proposals for new research should be informed by systematic reviews, and reports of new research should be set in the context of updated systematic reviews.

Trial Forge will aim to apply quality criteria when pointing to external resources and when collating individual studies. How to do this will form part of the initial work of Trial Forge, though it is likely that GRADE [32] (a system for grading the quality of evidence and the strength of recommendations, particularly for guidelines) will contribute importantly. Different approaches to presenting evidence will be explored using methods developed by the GRADE Working Group where appropriate, and the methods used to present the information will be informed by work done with, among others, the Cochrane Plain Language Summaries [33], the GRADE Summary of Findings tables [34, 35], and the DECIDE project (a project that aims to improve the way research information is presented in guidelines, http://www.decide-collaboration.eu). This presentation work will also be evaluated.

#### Step 3 - Strengthen the evidence base by creating a methodology research agenda

Step 3 will focus on strengthening the evidence base by providing a platform to highlight key areas of uncertainty, which would enable individuals and research groups to suggest ways in which the uncertainties could be addressed. For example, we know less about the effect of recruitment interventions aimed at recruiters than we do about those aimed at potential participants [7]. Recruiters play a hugely influential role and can have a substantial impact on recruitment [36, 37], but there remains uncertainty about how best to address the issues and concerns that recruiters face [36–45]. One way to help fill this gap (and others) may be through the availability of standard outlines for Studies Within A Trial (SWATs). The design of SWAT-1 is for site visits by the principal investigator to increase or maintain recruitment [46].

Publishing protocols for methodology research, which can then be embedded in other studies, makes it easier for research groups to become involved in filling evidence gaps. Much of the intellectual work around the appropriate methodology research already will have been done by the authors of the protocol. A database of outlines for SWATs is being developed to improve access to these ideas [47]. Step 3 of Trial Forge will produce SWATs as well as link people to initiatives such as the MRC-funded Systematic Techniques for Assisting Recruitment to Trials (START) programme (http://www.population-health.manchester.ac.uk/mrcstart/), which is developing a platform to evaluate recruitment interventions simultaneously across many trials.

Finally, where evidence does not yet exist, information about these gaps will be systematically directed to funding agencies for consideration in their prioritisation processes. In the meantime, Trial Forge will provide a repository for experience and knowledge from the community of trialists, trial managers, and others about interventions and approaches that they believed worked well in their settings. Trial Forge will thus provide support for electronically linked communities of practice (for example, through question and answer and discussion sections on its website) to facilitate sharing of knowledge and experiences, especially when rigorous evidence to inform decisions is lacking.

#### Step 4 - Collaborate to work through the methodology research agenda

A methodology research agenda will have been created in Step 3. Step 4 will encourage wide collaboration among methodologists, trialists, and other relevant stakeholders to tackle this research agenda. For some processes in the trial pathway (Fig. 1), the agenda will be substantial and very challenging. A single research group or trials unit is unlikely to have the skills, capacity, or interest to take on a whole agenda. By bringing research groups together around a shared agenda, Trial Forge will minimise unnecessary duplication, focus work on topics shown to be most in need of attention (with a recent survey of the priorities of UK Clinical Trials Unit Directors providing a good starting point [48]), and identify groups with the necessary expertise to do the work. For example, groups could work together to evaluate an intervention described in a SWAT. This collaboration between groups may happen naturally through direct contact but could be facilitated by Trial Forge, for example by having a coordinator identify potential links and encouraging collaboration.

#### Step 5 - Dissemination

The value of the expanded evidence base will be realised in Step 5: when Trial Forge has identified or generated an important result from, for example, an up-to-date systematic review of relevant methodology research, people who need to know about it should be informed efficiently. For example, if, as a result of including new trial data to the Cochrane review of interventions to improve retention in trials [8] meant that there was now clear evidence that a particular intervention was effective, Trial Forge would help to ensure that this information is disseminated efficiently to trialists. A variety of dissemination routes will be used, for example, electronic mailing lists, a Twitter feed, presentations at the UK Clinical Trials Units Directors’ meetings and training courses. Dissemination routes are likely to need to change over time and may well need to differ depending on the trial process being addressed. An underlying principle will be that simply publishing the findings in a journal article is unlikely to be sufficient to promote uptake. To maximise the impact of this methodology research, Trial Forge will use evidence from implementation research to promote clinical and professional behaviour change interventions [49]. This step of Trial Forge will also be evaluated.

The five steps in Trial Forge will be iterative, especially since many trial processes are linked and because suggestions for change in one area may have consequences for others. Trial Forge’s own processes will also be evaluated and modified over time as we and others learn from our experience of using the five steps to reduce gaps in knowledge about how best to design, conduct, analyse, and report trials. Once started, Trial Forge should produce a steady stream of methodology innovations that address trial process problems of recognised significance to people involved in trials. Importantly, work, and prestige will not be concentrated in one place or group but will be distributed across a collaborative network. Groups engaging with Trial Forge will be encouraged to build up their own portfolios of methodology work in areas that match their interests and expertise.

## Conclusion

Trial Forge aims to support active and regular engagement with people who design, conduct, analyse, and report trials in the UK and elsewhere. It will promote meaningful improvements in trial efficiency and greater potential for trials to improve health. Moreover, Trial Forge will support an informal network of interested trialists, who will meet virtually and occasionally in person to build capacity and knowledge in efficient trials design and conduct. It will aim to be the ‘go to’ website for summaries of what is known about trial methods research but also for a linked programme of applied methodology research that encourages people to collaborate to fill gaps in evidence.

Not all problems in trials need more methodology research. However, many aspects of trial design, conduct, analysis, and reporting could be subjected to research to identify the relative effects of alternative approaches and whether these aspects are scientific, methodological, or administrative; they all have uncertainties that could be addressed by research leading to greater evidence-based approaches than is currently the case. We believe that Trial Forge will maximise the effectiveness and efficiency of trials, increase the chances that they will produce reliable and robust answers, and minimise waste. Trialists share many of the same problems; Trial Forge is about working together to solve them.

## Abbreviations

COMET: Core Outcome Measures in Effectiveness Trials
DECIDE: Developing and Evaluating Communication Strategies to Support Informed Decisions and Practice Based on Evidence
GRADE: Grading of Recommendations Assessment, Development, and Evaluation
MRC: Medical Research Council
START: Systematic Techniques for Assisting Recruitment to Trials
SWAT: Studies Within A Trial
